# Inhibition of advanced glycation end products by red grape skin extract and its antioxidant activity

**DOI:** 10.1186/1472-6882-13-171

**Published:** 2013-07-12

**Authors:** Nattha Jariyapamornkoon, Sirintorn Yibchok-anun, Sirichai Adisakwattana

**Affiliations:** 1 Department of Pharmacology, Faculty of Veterinary Science, Chulalongkorn University, Bangkok, Thailand; 2 Research Group of Herbal Medicine for Prevention and Therapeutic of Metabolic diseases, Chulalongkorn University, Bangkok, Thailand; 3 Department of Nutrition and Dietetics, Faculty of Allied Health Sciences, Chulalongkorn University, Bangkok, Thailand

**Keywords:** Grape skin, Anthocyanin, Antioxidant, Antiglycation

## Abstract

**Background:**

The objective of the present study was to determine the phytochemical content and the protective effect of red grape skin extract (RGSE) against fructose-mediated protein oxidation. In addition, RGSE was screened for its potential as an antioxidant using various in vitro models.

**Methods:**

Antioxidant activity was measured by 2,2-diphenyl-1-picrylhydrazyl (DPPH), hydroxyl radical scavenging activity, superoxide radical scavenging activity, trolox equivalent antioxidant capacity, ferric reducing antioxidant power (FRAP), ferrous ion chelating power. The total phenols content was measured by Folin–Ciocalteu assay, the flavonoids content by the AlCl_3_ colorimetric method. Antiglycation activity was determined using the formation of AGE fluorescence intensity, N^ϵ^-(carboxymethyl)lysine, and the level of fructosamine. The protein oxidation was examined using the level of protein carbonyl content and thiol group.

**Results:**

The results showed that the content of total phenolics, flavonoids and total anthocyanins in RGSE was 246.3 ± 0.9 mg gallic acid equivalent/g dried extract, 215.9 ± 1.3 mg catechin equivalent/g dried extract, and 36.7 ± 0.8 mg cyanidin-3-glucoside equivalent/g dried extract, respectively. In the DPPH radical scavenging activity, hydroxyl radical scavenging activity, and superoxide radical scavenging activity, RGSE had the IC_50_ values of 0.03 ± 0.01 mg/ml, 5.40 ± 0.01 mg/ml, and 0.58 ± 0.01 mg/ml, respectively. In addition, RGSE had trolox equivalent antioxidant capacity assay (395.65 ± 1.61 mg trolox equivalent/g dried extract), ferric reducing antioxidant power (114.24 ± 0.03 mM FeSO_4_/g dried extract), and ferrous ion chelating power (3,474.05 ± 5.55 mg EDTA/g dried extract), respectively. The results showed that RGSE at different concentrations (0.031–0.500 mg/ml) has significantly inhibited the formation of AGEs in terms of the fluorescence intensity of glycated BSA during 4 weeks of study. The RGSE markedly decreased the level of fructosamine, which is directly associated with the reduction of AGE formation and N^ϵ^-(carboxymethyl)lysine (CML). The results demonstrated the significant effect of RGSE on preventing protein oxidative damages, including effects on the thiol and protein carbonyl oxidation.

**Conclusions:**

The present study revealed that RGSE would exert beneficial effects by virtue of its antioxidants and antiglycation. The findings could provide a new insight into the naturally occurring antiglycation properties of RGSE for preventing AGE-mediated diabetic complication.

## Background

Protein glycation is a non-enzymatic reaction that initiates from a complex cascade of several reactions between reducing sugar and free amino group, resulting in the formation of a reversible structure called a Schiff’s base [1]. Then, it undergoes rearrangements to the Amadori products that induce further oxidation, generating dicarbonyl compounds to form cross-linking fluorescent (e.g., pentosidine) and non-fluorescent adducts (e.g., CML) called advanced glycation end products (AGEs). AGEs, the unstable and irreversible product of glycation process, can react with other free amino group and lead to protein modification such as alternative protein half-life, immune system, and enzyme function, leading to physiopathological changes [2]. Many studies have revealed a vital role for protein glycation in the pathogenesis of age-related diseases, such as diabetes, atherosclerosis, end-stage renal disease, and neurodegenerative disease [2]. There has recently been much interest in using antiglycation for alleviating diabetic complications [3]. Aminoguanidine (AG), a hydrazine derivative, acts by trapping reactive carbonyl intermediates and blocking formation of Schiff base or AGEs [4]. However, it has shown serious side effects in type 2 diabetic nephropathy such as flu-like symptoms, gastrointestinal problems, and anemia [5]. Therefore, much effort has been extended in search of dietary plants and fruits that effectively inhibit AGE formation.

Red grape, *Vitis vinifera* is one of the most popular and widely cultivated fruits in the world. The skin of the red grape contains many active components, including flavonoids, anthocyanins, procyanidins, and the stilbene derivatives resveratrol. The major pharmacological properties of red grape skin and its constituents are anti-cancer [6], anti-diabetes [7], anti-obesity [8], anti-platelet aggregation [9], and anti-hypertension [10]. The preliminary observations indicate that grape skin and seed inhibit protein glycation in bovine serum albumin [11,12]. Anthocyanins are pigments, and mainly exist in grape skins. It has been shown that grape skin is able to act as a natural anthocyanin against mammalian intestinal α-glucosidase and pancreatic α-amylase [13-16]. However, there are no studies supporting the ability of grape skin in the prevention of diabetic complications by inhibiting the formation of AGEs, the level of fructosamine, protein oxidation, and non-fluorescent adducts. Therefore, it was interesting to investigate the effects of the RGSE against fructose-mediated non-enzymatic glycation and oxidation-dependent damages to BSA. In addition, the phytochemical contents and bioactivity of RGSE related to antioxidants including 1,1-diphenyl-2-pireyhydrazyl (DPPH) radical scavenging activity, trolox equivalent antioxidant capacity assay (TEAC), ferric reducing antioxidant power (FRAP) assay, hydroxyl radical scavenging activity (HRSA), and superoxide radical scavenging activity (SRSA), and metal chelating activity were also evaluated.

## Methods

### Chemicals

Bovine serum albumin (BSA), 2,2-diphenyl-1-picrylhydrazyl (DPPH), 2,2-azinobis3-ethylbenzothiazoline-6-sulfonic acid (Trolox), 2,4,6- tripyridyl-S-triazine (TPTZ), iron sulfate (FeSO_4_), xanthine, xanthine oxidase, 5,5′-dithiobisnitro benzoic acid (DTNB), nitroblue tetrazolium (NBT), 1-deoxy-1-morpholinofructose (DMF), 2,4-dinitrophenylhydrazine (DNPH), and L-cysteine were purchased from Sigma Chemical Co. (St. Louis, MO, USA). Fructose, Folin–Ciocalteu’s phenol reagent, and gallic acid were purchased from Fluka (St. Louis, MO, USA). N^ϵ^-(carboxymethyl) lysine (CML) test kit was purchased from Cell Biolabs Inc. (U.S.A). The dried powder of red grape skin extract (RGSE) (article no. 825F) was obtained from Breko GmbH Co. (Bremen, Germany).

### Phytochemical analysis

The RGSE (1 mg) was dissolved in distilled water (1 ml). The total polyphenolic and flavonoid content in RGSE was determined using Folin–Ciocalteu’s phenol reagent and aluminum chloride colorimetric method, respectively [12]. The total anthocyanin content in RGSE was determined using pH differential method [17]. The total polyphenolic, total flavonoid, and total anthocyanin content were expressed as mg gallic acid equivalent/g dried extract, mg catechin equivalent/g dried extract, and mg cyanidin-3-glucoside equivalent/g dried extract, respectively (n = 3).

### DPPH radical scavenging activity

RGSE was dissolved in phosphate buffered saline (PBS), pH 7.4. Antioxidant capacity was measured using the DPPH assay as described by a previous method [18]. Briefly, 100 μl of the solution containing RGSE dissolved in PBS (0.0125–0.100 mg/ml) was added to 100 μl of a DPPH solution (0.2 mM in ethanol) and incubated for 30 min at room temperature. The decrease in the solution absorbance was measured at 515 nm. The IC_50_ value was calculated from the plotted graph of % DPPH scavenging ability against the concentrations of the samples. Ascorbic acid was used as a positive control for this study.

### Trolox equivalent antioxidant capacity assay

Trolox equivalent antioxidant capacity assay (TEAC) of each sample was determined according to a method described [19]. The ABTS · ^+^ was generated by persulfate oxidation of ABTS by incubation at room temperature for at least 16 hours in darkness. ABTS^º+^ solution was diluted with phosphate buffer solution to absorbance values of 0.70 ± 0.02 at 734 nm. For measuring antioxidant capacity, 500 μl of the solution containing RGSE dissolved in PBS was added with 990 μl of ABTS · ^+^ solution. The decrease in the absorbance was measured at 734 nm after 6 min. Trolox was used as a positive control for this study. TEAC value was calculated from a standard curve by using trolox. TEAC value was expressed as mg of trolox equivalents per gram of dried extract.

### Ferric reducing antioxidant power (FRAP)

Ferric reducing antioxidant power (FRAP) was measured according to a previous method with slight modifications [20]. Briefly, FRAP solution was mixed with 10 ml of 0.3 M sodium acetate buffer solution (pH 3.6), 1 ml of 10 mM 2,4,6- tripyridyl-S-triazine (TPTZ) in 40 mM HCl, and 1 ml of 20 mM FeCl_3_. The solution containing RGSE dissolved in PBS (0.2 ml) was added with 1.8 ml of FRAP solution as oxidizing reagent and incubated for 30 min at room temperature. The increase in the solution absorbance was measured at 593 nm. FRAP value was calculated from a standard curve by using FeSO_4_. FRAP value was expressed as mM of FeSO_4_ equivalents per gram of dried extract.

### Hydroxyl radical scavenging activity

Hydroxyl radical scavenging activity (HRSA) was measured by a previous method with minor modifications [21]. The reaction mixture was done by adding 33 μl of 17 mM 2-deoxy2-ribose, 33 μl of the solution containing RGSE dissolved in PBS (0.5–30 mg/ml), 33 μl of 1.2 mM EDTA, 67 μl of 0.3 mM FeCl_3_, 33 μl of 34 mM hydrogen peroxide (H_2_O_2_), and 67 μl of 0.6 mM ascorbic acid. The reaction was performed at 37°C for 1 h. Thereafter, 333 μl of 1% (w/v) thiobarbituric acid (TBA) and 333 μl of 2.8% (w/v) trichloroacetic acid (TCA) were added to the mixture and were incubated at 100°C for 15 min. After cooling, the absorbance was measured at 532 nm against a blank containing deoxyribose and buffer. The IC_50_ value was calculated from the plotted graph of % radical scavenging against the concentrations of the samples. Trolox was used as a positive control for this study.

### Superoxide radical scavenging activity

The measurement of superoxide radical scavenging activity (SRSA) was done according to a previous method with slight modifications [22]. In brief, 50 μl of the solution containing RGSE dissolved in PBS (0.125–5.00 mg/ml), 500 μl of 0.30 mM xanthine, 250 μl of 0.15 mM NBT, 250 μl of 0.60 mM EDTA, and 50 μl of xanthine oxidase (0.05 unit/ml) were mixed and placed in the wells of a microplate. After incubation for 40 min at 37°C, the absorbance was measured at 560 nm. The IC_50_ value was calculated from the plotted graph of %radical scavenging against the concentrations of the samples. Trolox was used as a positive control for this study.

### Ferrous ion chelating power

Ferrous ion chelating power (FICP) was measured by 2,2′-bipyridyl competing assay according to a previous study with slight modifications [23]. In short, 0.25 ml of FeSO_4_ solution (1 mM) and an equal volume of the solution containing RGSE dissolved in PBS were mixed and 1 ml of 0.1 M Tris–HCl buffer (pH 7.4) and 1 ml of 2,2′-bipyridyl solution (0.1% in 0.2 M HCl) were added to the mixture, together with 0.4 ml of 10% (w/v) hydroxylamine-HCl and 1.5 ml of ethanol. After filling the reaction mixture up to 5 ml with distilled water, the absorbance of the solution was measured at 562 nm. FICP value was calculated from a standard curve by using EDTA. FICP value was expressed as mg of EDTA equivalents per gram of dried extract.

### In vitro glycation of bovine serum albumin

The glycated BSA formation was undertaken in accordance with a previous method [24]. Briefly, BSA (10 mg/ml) was incubated with 1.1 M fructose in 0.1 M phosphate buffered-saline (PBS), pH 7.4 containing 0.02% sodium azide in darkness at 37°C for 1, 2, 3, and 4 weeks. Before incubation, the solution containing RGSE dissolved in PBS (0.031–0.500 mg/ml) was added to the mixtures. The glycated BSA formation was determined using fluorescent intensity at an excitation wavelength 355 nm and emission wavelength 460 nm. Aminoguanidine (AG) was used as a positive control for this study.

### Determination of Fructosamine

After 1, 2, 3, and 4 weeks of incubation, the concentration of fructosamine, the Amadori product, was measured by NBT assay [24]. Briefly, glycated BSA (10 μl) was incubated with 90 μl of 0.5 mM NBT in 0.1 M carbonate buffer, pH 10.4 at 37°C. The absorbance was measured at 530 nm at 10 and 15 min time points. The concentration of fructosamine was calculated compared to 1-deoxy-1-morpholino-fructose (1-DMF) as the standard.

### Determination of protein carbonyl content

After 1, 2, 3, and 4 weeks of incubation, the carbonyl group in glycated BSA, a marker for protein oxidative damage, was assayed according to a previous method of Levine and colleagues with minor modifications [24]. Briefly, 800 μl of 10 mM DNPH in 2.5 M HCl was added to 200 μl of glycated samples. After 1 h incubation in the dark, 1 ml of 20% (w/v) TCA was used for protein precipitation (5 min on ice) and then centrifuged at 10,000 g for 10 min at 4°C. The protein pellet was washed with 500 μl of ethanol/ethyl acetate (1:1) mixture 3 times and resuspended in 500 μl of 6 M guanidine hydrochloride. The absorbance was measured at 370 nm. The carbonyl content of each sample was calculated based on the extinction coefficient for DNPH (ϵ = 22,000 M^-1^cm^-1^). The results were expressed as nmol carbonyl/mg protein.

### Determination of thiol group

After 1, 2, 3, and 4 weeks of incubation, the free thiols in glycated samples were measured by Ellman’s assay with minor modifications [24]. Briefly, 70 μl of glycated samples were incubated with 130 μl of 5 mM DTNB in 0.1 M PBS, pH 7.4 at 25°C for 15 min. The absorbance of samples was measured at 410 nm. The concentration of free thiols was calculated from L-cysteine standard and expressed as nmol/mg protein.

### Determination of N^ϵ^-(carboxymethyl) lysine

After 4 weeks of incubation, N^ϵ^-(carboxymethyl) lysine (CML), a major antigenic AGE structure, was determined using enzyme linked immunosorbant assay (ELISA) kit. According to the manufacturer’s protocol, the glycated samples were diluted to final concentration of 1 μg/ml (10,000-fold dilution) before used in the assay. Each diluted sample (100 μl) was incubated in the 96-well protein binding plate at 37°C for at least 2 h. After washing with PBS, an assay diluent was added and further incubated for 2 h at room temperature on an orbital shaker. Three washes with wash buffer were needed before incubating for 1 h each with anti-CML antibody and with secondary antibody-HRP conjugate. The substrate solution (100 μl) was added for 20 min before adding stop solution in an equal volume. The absorbance of samples was measured immediately at 450 nm and compared with the absorbance of CML-BSA standard providing in the assay kit.

### Statistical analysis

Data were presented as mean ± standard error of mean (S.E.M) for N = 3. Data were analyzed using one-way analysis of variance (ANOVA) and Tukey’s HSD test with p < 0.05 were considered significant.

## Results

### Phytochemical analysis of RGSE

Our determination revealed that the content of total phenolic compounds in RGSE was 246.3 ± 0.9 mg gallic acid equivalent/g dried extract. In the meanwhile, the content of total flavonoids in RGSE was 215.9 ± 1.3 mg catechin equivalent/g dried extract. In addition, the content of total anthocyanins in RGSE was determined to be 36.7 ± 0.8 mg cyanidin-3-glucoside equivalent/g dried extract.

### Antioxidant activity of RGSE

The effects of RGSE on different antioxidant capacities are shown in Table 1. On the DPPH assay, RGSE had significant radical scavenging activity with increasing concentration in the range of 0.0125–0.1000 mg/ml. The IC_50_ values of RGSE and ascorbic acid were found to be 0.03 ± 0.01 mg/ml and 0.01 ± 0.01 mg/ml, respectively. The results showed that RGSE had 3.0-times less potency than ascorbic acid. According to the results from TEAC, RGSE had antioxidant activity of 395.65 ± 1.61 mg trolox equivalent/g dried extract. FRAP assay determines the reducing ability of an antioxidant reacting with a ferric tripyridyltriazine (Fe^3+^–TPTZ) complex and producing a colored ferrous tripyridyltriazine (Fe^2+^–TPTZ). RGSE had FRAP value of 114.24 ± 0.03 mM FeSO_4_/g dried extract. The hydroxyl radical is one of representative reactive oxygen species generated in the body. The results showed that the IC_50_ values of RGSE and trolox were 5.40 ± 0.01 and 2.16 ± 0.06 mg/ml, respectively. In addition, RGSE had a scavenging activity on the superoxide radicals in a concentration-dependent manner with the IC_50_ value of 0.58 ± 0.01 mg/ml, while the IC_50_ value of trolox was found to be 0.32 ± 0.01 mg/ml. The results indicated that RGSE had 1.81-times less potency than trolox. In addition, RGSE were assayed for its Fe^2+^ chelating power, and this activity was compared with the chelating activity of the synthetic metal chelator EDTA. The results showed that RGSE had the FICP value of 3,474.05 ± 5.55 mg EDTA/g dried extract.

**Table 1:** **Antioxidant activity of RGSE including DPPH radical scavenging activity**, **TEAC**, **FRAP**, **HRSA**, **SRSA**, **and FICP**

	**Antioxidant activity**					
	**DPPH**	**TEAC**	**FRAP**	**HRSA**	**SRSA**	**FICP**
**RGSE**	0.03 ± 0.01	395.65 ± 1.61	114.24 ± 0.03	5.40 ± 0.01	0.58 ± 0.01	3,474.05 ± 5.55
**Ascorbic acid**	0.01 ± 0.01	-	-	-	-	-
**Trolox**	-	-	-	2.16 ± 0.06	0.32 ± 0.01	-

Data are expressed as mean ± S.E.M, n = 3. DPPH radical scavenging activity, Hydroxyl radical scavenging activity (HRSA), and Superoxide radical scavenging activity (SRSA) are expressed as the IC_50_ value (mg/ml). TEAC, FRAP, and FICP are expressed as mg trolox/gram dried extract, mM FeSO_4_/gram dried extract, and mg EDTA/gram dried extract, respectively.

### The effects of RGSE on AGEs formation

**Figure 1:**
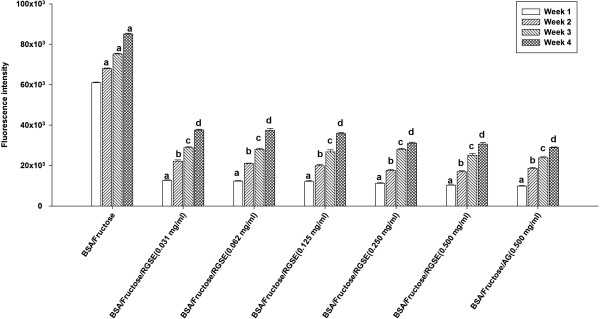
**The effects of red grape skin extract (RGSE) on formation of fluorescent advanced glycation end products (AGEs) in BSA incubated with fructose.** Each value represents the mean ± SE (n = 3). ^a^p < 0.05 when compared to BSA/fructose at week 1; ^b^p < 0.05 when compared to BSA/fructose at week 2; ^c^p < 0.05 when compared to BSA/fructose at week 3; ^d^p < 0.05 when compared to BSA/fructose at week 4.

As shown in Figure 1, the formation of AGEs was monitored weekly by measuring fluorescence intensity of the BSA-fructose solutions. When BSA was incubated with fructose, the significant increase in fluorescence intensity was observed during 4 weeks of the experiment. After the RGSE was added to reaction media containing BSA/fructose system, the fluorescence intensity was significantly decreased in a concentration-dependent manner throughout the study period. At week 4 of incubation, the percentage inhibition of AGEs formation by RGSE (0.031–0.500 mg/ml) was 55.23% to 63.52%, respectively. A significant inhibition of AGEs formation (73.3%) was observed in fructose-induced glycated BSA plus AG (0.5 mg/ml).

### The effects of RGSE on the level of fructosamine

The effects of RGSE on the level of fructosamine are shown in Table 2. The level of fructosamine in BSA/fructose increased markedly throughout the 4 weeks of the experiment. In contrast, the addition of RGSE together with BSA/fructose significantly suppressed the generation of fructosamine at weeks 1 to 4. At the end of study, RGSE at the concentration of 0.062–0.500 mg/ml significantly reduced the level of fructosamine, by approximately 4.0%–10.5%., whereas AG (0.500 mg/ml) decreased the level of fructosamine by 15.4%.

**Table 2:** **The effects of RGSE on the level of fructosamine in BSA**/**fructose system**

**Experimental groups**	**Fructosamine (mM)**			
	**Week 1**	**Week 2**	**Week 3**	**Week 4**
BSA/fructose	1.69 ± 0.03	2.05 ± 0.04^a^	2.30 ± 0.05^a^	2.47 ± 0.06^a^
BSA/fructose/RGSE (0.031 mg/ml)	1.57 ± 0.02	1.99 ± 0.05	2.11 ± 0.06^c^	2.40 ± 0.08
BSA/fructose/RGSE (0.062 mg/ml)	1.46 ± 0.05^a^	1.85 ± 0.02^b^	2.00 ± 0.04^c^	2.37 ± 0.01^d^
BSA/fructose/RGSE (0.125 mg/ml)	1.48 ± 0.01^a^	1.69 ± 0.05^b^	2.09 ± 0.03^c^	2.35 ± 0.03^d^
BSA/fructose/RGSE (0.250 mg/ml)	1.44 ± 0.02^a^	1.69 ± 0.09^b^	2.05 ± 0.04^c^	2.28 ± 0.07^d^
BSA/fructose/RGSE (0.500 mg/ml)	1.35 ± 0.05^a^	1.66 ± 0.04^b^	2.00 ± 0.06^c^	2.21 ± 0.04^d^
BSA/fructose/AG (0.500 mg/ml)	1.31 ± 0.02^a^	1.60 ± 0.06^b^	2.09 ± 0.02^c^	2.09 ± 0.06^d^

Results are expressed as mean ± SEM (n = 3). ^a^p < 0.05 when compared to BSA/fructose at week 1; ^b^p < 0.05 when compared to BSA/fructose at week 2; ^c^p < 0.05 when compared to BSA/fructose at week 3; ^d^p < 0.05 when compared to BSA/fructose at week 4.

### The effects of RGSE on protein oxidation

The determination of carbonyl content and thiol groups was used in order to assess the protein oxidation that occurred during the process of glycation. As shown in Table 3, the carbonyl content of glycated BSA was significantly increased during the experimental period, whereas BSA/fructose incubated with RGSE (0.062–0.500 mg/ml) significantly attenuated an increase in protein carbonyl content of BSA. At week 4, compared to BSA/fructose, the percentage reduction of carbonyl content by RGSE at concentration of 0.062–0.500 mg/ml was 37.7% to 41.7%, whereas that by AG was 45.1%.

**Table 3:** The effects of RGSE on carbonyl content in BSA/fructose system

**Experimental groups**	**Protein carbonyl content (nmol/mg protein)**			
	**Week 1**	**Week 2**	**Week 3**	**Week 4**
BSA/fructose	1.49 ± 0.54	2.56 ± 0.06^a^	3.48 ± 0.05^a^	4.44 ± 0.06^a^
BSA/fructose/RGSE (0.031 mg/ml)	1.21 ± 0.02^a^	1.78 ± 0.06^b^	2.42 ± 0.01^c^	2.92 ± 0.02^d^
BSA/fructose/RGSE (0.062 mg/ml)	1.26 ± 0.04^a^	1.79 ± 0.06^b^	2.41 ± 0.02^c^	2.76 ± 0.05^d^
BSA/fructose/RGSE (0.125 mg/ml)	1.13 ± 0.03^a^	1.59 ± 0.02^b^	2.28 ± 0.03^c^	2.76 ± 0.05^d^
BSA/fructose/RGSE (0.250 mg/ml)	1.07 ± 0.03^a^	1.49 ± 0.07^b^	2.34 ± 0.02^c^	2.72 ± 0.02^d^
BSA/fructose/RGSE (0.500 mg/ml)	0.99 ± 0.01^a^	1.45 ± 0.02^b^	2.24 ± 0.04^c^	2.59 ± 0.02^d^
BSA/fructose/AG (0.500 mg/ml)	0.87 ± 0.01^a^	1.73 ± 0.04^b^	2.05 ± 0.03^c^	2.31 ± 0.02^d^

Results are expressed as mean ± SEM (n = 3). ^a^p < 0.05 when compared to BSA/fructose at week 1; ^b^p < 0.05 when compared to BSA/fructose at week 2; ^c^p < 0.05 when compared to BSA/fructose at week 3; ^d^p < 0.05 when compared to BSA/fructose at week 4.

The effects of RGSE on the oxidation of protein thiols are shown in Table 4. When BSA was incubated with fructose, the level of thiol groups had continuously decreased throughout the experimental period. When comparing with BSA/fructose at week 1, BSA/fructose had a 70.1% reduction in the level of thiol groups at week 4. In the meantime, there was a significant improvement in the level of thiol groups after addition of RGSE (0.062–0.500 mg/ml) as well as AG (0.500 mg/ml). The findings showed that the percentage improvement of thiol groups by RGSE was between 27.6% and 50.4%, whereas AG significantly prevented (57.9%) the depletion of protein thiol groups.

**Table 4:** The effects of RGSE on the level of thiol group in BSA/fructose system

**Experimental groups**	**Thiol group (nmol/mg protein)**			
	**Week 1**	**Week 2**	**Week 3**	**Week 4**
BSA/fructose	2.31 ± 0.06	1.71 ± 0.10^a^	1.12 ± 0.10^a^	0.69 ± 0.14^a^
BSA/fructose/RGSE (0.031 mg/ml)	2.29 ± 0.08	2.04 ± 0.09^b^	1.57 ± 0.09^c^	0.83 ± 0.17
BSA/fructose/RGSE (0.062 mg/ml)	2.33 ± 0.09	2.15 ± 0.11^b^	1.61 ± 0.11^c^	0.88 ± 0.08^d^
BSA/fructose/RGSE (0.125 mg/ml)	2.51 ± 0.07^a^	2.16 ± 0.06^b^	1.53 ± 0.07^c^	0.97 ± 0.15^d^
BSA/fructose/RGSE (0.250 mg/ml)	2.41 ± 0.13^a^	2.26 ± 0.08^b^	1.56 ± 0.08^c^	0.94 ± 0.07^d^
BSA/fructose/RGSE (0.500 mg/ml)	2.60 ± 0.10^a^	2.37 ± 0.10^b^	1.63 ± 0.10^c^	1.04 ± 0.12^d^
BSA/fructose/AG (0.500 mg/ml)	2.51 ± 0.09^a^	2.35 ± 0.07^b^	1.59 ±0.09^c^	1.09 ± 0.09^d^

Results are expressed as mean ± SEM (n = 3). ^a^p < 0.05 when compared to BSA/fructose at week 1; ^b^p < 0.05 when compared to BSA/fructose at week 2; ^c^p < 0.05 when compared to BSA/fructose at week 3; ^d^p < 0.05 when compared to BSA/fructose at week 4.

### The effect of RGSE on CML formation

**Figure 2:**
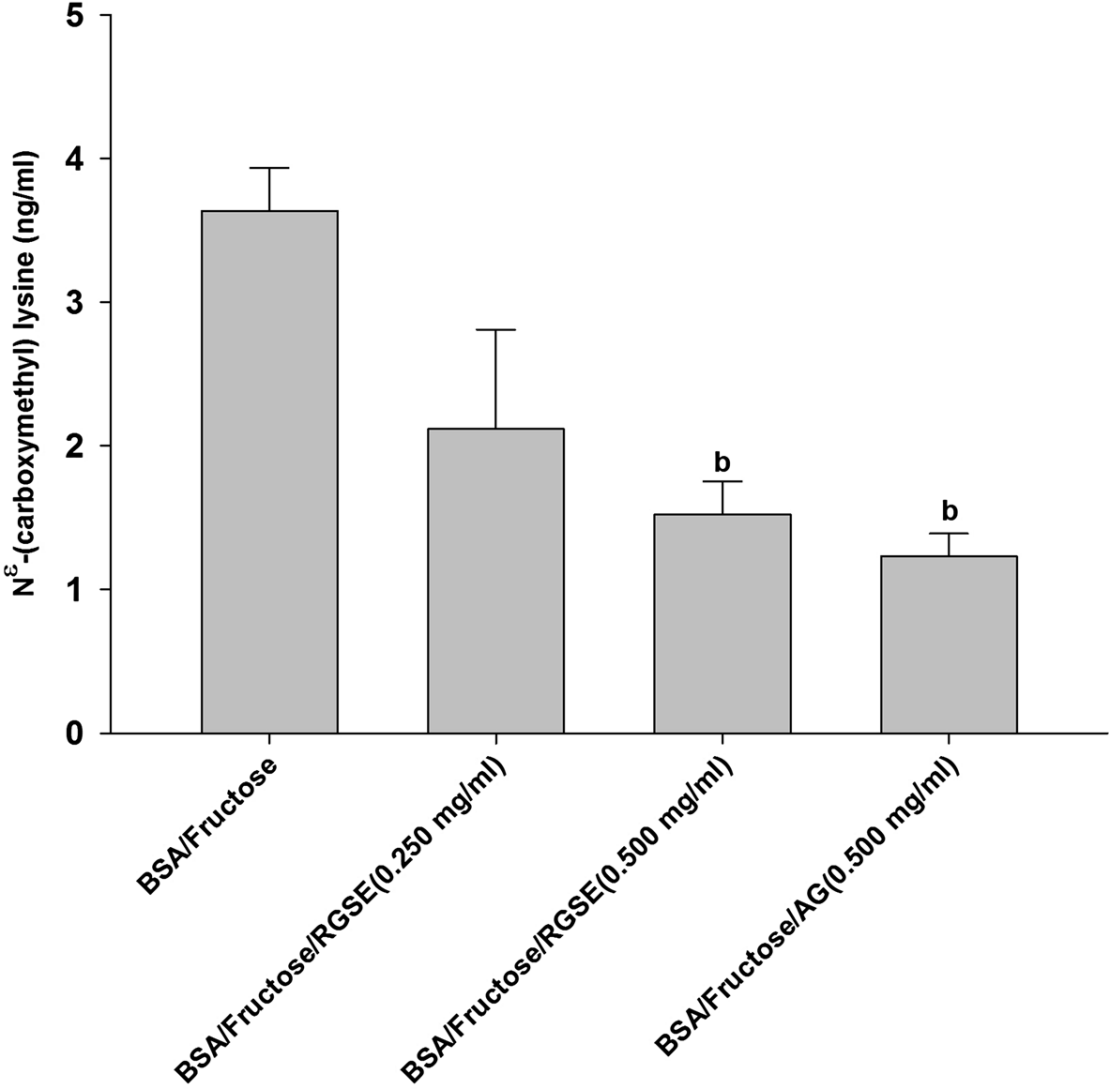
**The effects of red grape skin extract (RGSE) on the level of N**^**ϵ**^**-(carboxymethyl) lysine (CML) in BSA incubated with fructose after 4 weeks of incubation.** Each value represents the mean ± SEM (n = 3). ^b^p < 0.05 compared to BSA/ Fructose.

CML has been used as a biomarker for the formation of non-fluorescent AGE. As shown in Figure 2, the RGSE (0.250 and 0.500 mg/ml) decreased 41.7% and 58.1% of CML formation when compared to BSA/fructose. In the group of AG (0.500 mg/ml), the formation of CML was decreased by 72.5%, as compared to BSA/fructose.

## Discussion

The glycation process causes various types of protein and chemical modifications, resulting in the generation of irreversible heterogeneous byproducts termed advanced glycation end products (AGEs). The accumulation of AGEs plays a primary role in the aging process as well as the pathogenesis of age-related disorders including Alzheimer’s disease and diabetic complications [25,26]. In recent decades, over consumption of high-fructose diets has dramatically increased and has been linked to an increase in obesity and diabetic complications. Nowadays, there has been much concern regarding the important role of dietary fructose in the development of metabolic diseases. In the context of intracellular glycation, the rate for fructose is faster than that of glucose [27,28]. Because of the faster rate of the reactive glycolytic intermediates in the formation of AGEs, fructose and its metabolites are believed to be important precursors in the intracellular formation of AGEs. In the early stage of glycation, unstable Schiff’s bases are formed and turned into Amadori products such as fructosamine, which is clinically used as an indicator for short term control of blood sugar in diabetic patients [29]. The reduction of fructosamine, therefore, is a therapeutic way to delay incident vascular complications [30]. In addition, the production of N^ϵ^-(carboxymethyl)lysine (CML) is one of the best characterized compounds of advanced glycation end products which are generated either from oxidative breakdown of Amadori products or polyol pathway mediated by α-oxoaldehydes such as glyoxal, methylglyoxal, and 3-deoxyglucosone [31]. Aside from the formation of AGEs, reactive carbonyl intermediaries and protein carbonyl derivatives also cause protein modifications that are particularly prone to oxidative reaction to amino acid such a cysteine. The reactive oxygen species are generated during glycation and glyoxidation and are able to oxidize side chains of amino acid residues in protein to form carbonyl derivatives and also diminish an oxidative defense of protein by decreasing thiol groups [32,33]. Thus, these phenomena are reflective of high oxidative stress, protein oxidative damage, and formation of AGEs, which is the direct reflection of excess of free radical generation.

Based on the fluorescence property, we studied the influence of RGSE on the formation of total AGEs. Our results demonstrated that RGSE efficiently inhibited AGEs formation. Furthermore, RGSE also reduced the level of fructosamine and the formation of N^ϵ^-(carboxymethyl)lysine (CML) associated with decreased formation of AGEs. Consequently, significant elevation of protein carbonyl content and oxidation of thiols in BSA were observed when the protein was glycated by fructose. In contrast, when RGSE was added to the same systems, it significantly suppressed these processes. Several biochemical mechanisms of anti-glycation reactions have recently been proposed [3]. During the early stage of glycation, Schiff bases are prone to oxidation, generating free radicals, reactive carbonyl groups and the formed AGEs. Scavenging hydroxyl radicals and superoxide radicals can alleviate oxidative stress and reduce the generation of reactive carbonyl compounds [3]. In addition, transition metal also catalyzes auto-oxidation of glucose and further generates reactive carbonyl compounds to form AGEs. Thus, metal chelators may retard the process of AGEs by preventing further oxidation of Amadori products and metal-catalyzed glucose oxidation [34].

It has been reported that many antioxidant-containing foods can scavenge free-radicals generated during the glycation process as well as prevent reducing sugars and Amadori products from self-oxidation, leading to the inhibition of AGE formation [35]. In the present study, various methods of accessing antioxidant capacities have been used for RGSE. DPPH is widely used to evaluate the free radical scavenging abilities of phytochemical compounds in vitro. FRAP assay has been used extensively to evaluate the ability of edible plants to reduce ferric ions, reflecting their ability to decrease reactive oxygen species (ROS) [36]. TEAC assay has been used for assessing the capacity of edible plants to scavenge ABTS radicals [37]. The HRSA and SRSA have been applied to investigate the abilities of antioxidants to scavenge hydroxyl and superoxide radicals [38]. From the results obtained in the present study, RGSE showed potent antioxidant properties. According to the abovementioned antiglycation mechanisms, RGSE may inhibit AGE formation by decreasing the ROS formation or by scavenging the ROS formed in vitro by auto-oxidation of sugars and/or oxidative degradation of Amadori products. However, the antioxidant activity of RGSE might not be the only reason for explanation of the mechanism of antiglycation. Other mechanisms of antiglycation have been proposed, such as breaking the cross-linking structures in the formed AGEs and inhibiting the formation of late-stage Amadori products. Further comprehensive studies of RGSE are required to evaluate the antiglycation mechanisms described above.

Phenolic compounds, flavonoids and anthocyanins are constituents of many edible plants, and they are of current research interest because of their health-promoting effect as antioxidants. The phytochemical analysis of red grape skin reveals the presence of phenolic compounds such as proanthocyanidins, ellagic acid, myricetin, quercetin, rutin, kaempferol, trans-resveratrol, cyanidin-3-glucoside, delphinidin-3-O-glucoside (myrtillin), petunidin-3-O-glucoside, peonidin-3-O-glucoside, malvidin-3-O-glucoside [39,40]. Furthermore, grape seeds are a rich source of polyphenols, which are characterized by a variety of properties, such as antibacterial and antioxidant activities [41,42]. Significant variations in the levels of total phenolic compounds, flavonoids, and anthocyanins in skins and seeds from different varieties of red grapes have previously been reported [41-43]. Among 21 different cultivars, there are 45 anthocyanins, 28 flavonols, 8 flavan-3-ols, 9 cinnamic acids, 5 benzoic acids, 5 ellagic acids and 2 stilbenes detected in all grape skins [41].

The phenolic compounds, flavonoids and anthocyanins exhibit considerable free radical scavenging activities and metal ion chelating properties [44-46]. Most antiglycation agents from the edible plants have been reported to possess phenolic compounds and flavonoids [47-49]. It has been reported that inhibitory activity of flavonoids against protein glycation was strongly related to their scavenging effect on free radicals derived from the glycoxidation process [50]. Our findings indicate that RGSE has high phenolic compounds, flavonoids and anthocyanin content. According to the abovementioned studies, it can be assumed that phenolic compounds and flavonoids in the RGSE may contribute to the antioxidant activity and antiglycation.

## Conclusion

In conclusion, RGSE has potent inhibitory effects on protein glycation and oxidation-dependent damages to BSA. Furthermore, the results suggest that RGSE acts as an antioxidant with suppressing effect on the formation of AGEs. The findings may lead to the possibility of using RGSE for preventing AGE-mediated diabetic complications.

## Abbreviations

AGEs: Advanced glycation end products; CML: N^ϵ^-(carboxymethyl) lysine; BSA: Bovine serum albumin; RGSE: Red grape skin extract; AG: Aminoguanidine; TEAC: Trolox equivalent antioxidant capacity assay; FRAP: Ferric reducing antioxidant power; HRSA: Hydroxyl radical scavenging activity; SRSA: Superoxide radical scavenging activity; FICP: Ferrous ion chelating power.

## Competing interests

The authors declare that they have no competing interests.

## Authors’ contributions

SA and SY were responsible for conception and design, drafted the manuscript and revised it critically for important intellectual content. NJ conducted the experiments, organized the data analysis, and interpretation of data. All authors contributed to the drafting of the manuscript and agreed on the final version of the manuscript.

## Pre-publication history

The pre-publication history for this paper can be accessed here:

http://www.biomedcentral.com/1472-6882/13/171/prepub
